# Family Group Conferences as a Shared Decision-Making Strategy in Adults Mental Health Work

**DOI:** 10.3389/fpsyt.2021.663288

**Published:** 2021-07-13

**Authors:** Shulamit Ramon

**Affiliations:** Department of Allied Health, Midwifery and Social Work, University of Hertfordshire, Hatfield, United Kingdom

**Keywords:** family decision making strategy, adult mental health, participation process, implementation process, evaluation

## Abstract

Family Group conferences (FGC) provide a system by which a client and their family reach jointly key intervention decisions, from a number of options proposed by professionals. The system originated in child protection social work.

Conceptually FGC is based on the assumption that the family is potentially a supportive social system for an individual with a variety of difficulties, including mental ill health. Reaching a family network agreement can lead to long term positive outcomes in self-confidence and social relationships. This strategy of shared decision making (SDM) can re-unite the family around the client's needs and wishes. It fits well the strengths based and the recovery-oriented approaches to mental ill health.

**Methodologically**, this article provides a narrative review of existing empirical research about FGC in the context of adult mental health. In addition, two community case studies consisting of videos of a mother experiencing mental ill health and a daughter are analysed in terms of their **subjective** experience of the FGCs they were involved in, and looks at both the process and the outcomes of FGCs.

The **key findings** demonstrate a high level of satisfaction **from participating** in the FGC meeting, while the evidence pertaining to the outcomes is inconclusive. Only very few systematic review studies, or comparative studies of different approaches to family decision making, exist, and there are no studies which offer cost effectiveness analysis.

**Discussion:** The observed gap between the satisfaction from the process of FGC by the participants vs. the inconclusive outcomes relates to the implementation phase, in which the decisions made by the family are tested. Evaluating FGC processes and outcomes is complex. A systematic and comprehensive research of the implementation process is missing at this stage.

**In conclusion**, FGC is a promising strategy of SDM in adult mental health. The research evidence indicates the need for further exploration of its implementation process, evaluative methodology and methods.

## Introduction

This narrative review is aimed to respond to the following questions:

1. What is FGC and what does it offer to adults facing mental ill health challenges and their families?
2. Is FGC a shared decision making strategy?
3. Key existing empirical research on FGC with this group of adults
3.i. Methodology and methods
3.ii. Key findings concerning processes
3.iii. Key findings concerning outcomes
4. Future challenges for researching FGC with this group.

## Background

FGC (Family Group Conferences) or FGDM (Family group decision making; its Dutch name) is a system in which key care issues of an individual are sorted out by calling a family meeting to reach jointly relevant decisions and their implementation action plan. The strategy was developed initially in New Zealand, as part of an attempt to reduce the increasing number of Maori children taken into care, roughly based on the Maori's problem solving strategy by a meeting of their elders, to which relevant others are invited too (1). FGC has been practiced in social care, especially in the context of child protection, across English-speaking countries, but also in other countries (such as the Netherlands) and with other issues, such as mental health (2), restorative justice (Restorative Works 2019 Year Review), adults of working age and older people's domestic abuse (3). Edwards and Parkinson's (4) book provides an overview of FGC in different areas. A key aim of this article is to examine the shared decision- making (SDM) component of FGC with adults experiencing mental ill health, its process and outcomes through analysing the existing evidence from empirical studies of FGC with this group.

SDM is by now more widely perceived as a useful component of supporting this group, alongside a greater emphasis on the contribution of family members (some of whom are acting as informal carers). Therefore, we need to ask how productive and satisfactory are the FGC process and outcomes from the perspectives of the index client, family members, and service providers.

The unique features of FGC include:

1. Professionals (often social workers, but not necessarily so) working with the family have a key role in suggesting the FGC, proposing potential solution scenarios that are likely to resolve the difficulties faced by the index client and the family for the family group to consider, and to map the support for the implementation of the agreed plan proposed by the family at the end of the initial FGC meeting.
2. A professional service provider initiates the request to offer an FGC and appoints an independent co-ordinator. The co-ordinator is central to preparing the family meeting, recruiting different family members to participate in the meeting, at times in the context of enduring poor relationships which raise doubts about the option of finding a shared solution. The co-ordinator's withdrawal after the preparatory phase signals to the family the trust in its ability to select a valid solution and an effective implementation plan.
3. Empowering the family and the index client by giving them the opportunity through the FGC to reach key shared decisions concerning the index client's future and the contribution of family members to the implementation plan.
4. The ability of the family to come up with an agreed plan within one meeting, despite past strained relationships, functioning difficulties, and doubts concerning the future.
5. Advocates for people who could not come, or who were reluctant to do so, may join the family meeting at the request of the person they are advocating for.
6. The key role of follow up meetings (usually 1–2 within 3–12 months) to ensure the implementation of the plan.

This model is attractive in terms of its potential to secure positive effect, thus saving efforts, time, funding, reducing and resolving difficulties which significantly affect family members' lives. There are variations between different countries as to whether co-ordinators are independent professionals (e.g., UK) or prominent community members who are not professionals (e.g., the Netherlands), whether the professional who proposed potential solutions scenarios joins the meeting for the phase of explaining the scenarios or just provides a written summary, and proceedings as to what to do when the index client refuses to attend the meeting and/or its evaluation.

## Conceptual Framework

The FGC strategy combines more than one conceptual strand. The family is understood to be a social network likely to be supportive of individual members facing difficulties which impede their functioning. The FGC has been constructed to enable families to do so with a specific significant problem. It is expected that family members who do not act as carers would be asked to invest time and energy in supporting the index client. Existing evidence that carers' psychological, physical and economic viability might be negatively impacted by being a carer lands support to this request.

The view of the family as a system, in which each individual depends on the whole family, is an integral part of the underlying assumptions of the approach. Any positive change is significant in contributing to the reduction of tension within the family system, even if the index client does not return to a good level of functioning.

Different cultures vary in the place and power given to families. Hence cultural competence in FGC needs to be secured prior to applying it to each culture. The article by Barn and Das (5) provides a useful contribution concerning this theme. The authors highlight that in order to prevent the othering of the members of a minority group who may also be unsure of an initiative that comes from the majority's culture. Cultural competence requires knowledge of the history and culture of the group the family comes from, their preferences concerning issues such as the place of elders, use of language, and type of food preferred to be served in the family meeting. Barn and Das conducted an empirical research into how 12 managers and 8 co-ordinators of FGC projects in London approached this issue, collecting the evidence from a focus group and profile questionnaires. The findings highlighted that the participants attempt to find out at the referral point what is the cultural background, and then aim to provide ethnic matching, and if possible also matching in terms of language, gender and religion. If necessary, they pare co-ordinators with interpreters, and have bi-lingual co-ordinators.

### Attention to Mental Ill Health Challenges Within FGC With Adults

FGC is based on the assumption that in principle the family and its dynamics can be a powerful tool for reaching key decisions concerning its members and in implementing these decisions. It is furthermore assumed that these two components play a part even when one member of the family, or more than one, has experienced difficulties, such as mental ill health challenges. Shame, guilt, blame, bitterness, and unhappiness, typify the experience of many service users and their family members/carers where an identified mental ill health difficulty exists (6).

### Contextualising SDM and FGC in the Current Mental Health System

The development of community mental health services, and the considerable reduction in the place of institutionalised care for people experiencing mental ill health that has taken place gradually since the second half of the twentieth century, highlighted the fact that this group does not need to be segregated from society (7). With the success of rehabilitative options, such as being in employment (8), came also a re-evaluation of the abilities of members of this group.

Since the 1960s it is largely accepted that a mental health crisis has the potential to provide an opportunity to develop positive new options, hence need not be perceived only as harmful (9). This perspective relates to the definition of a crisis as an imbalance between arising difficulties and the resources necessary for resolving them (9). The strengths of the person, and of their family members, are part of these resources.

This logic has been taken further in the more recently developed concept of Posttraumatic Growth (10) which has highlighted that Posttraumatic Stress Disorder is not an end point, and that identifying positive lessons in traumatic experiences is both possible and desirable (11). It can also be acquired through a learning process with providers trained specifically to mentor the process of applying PTG, called Expert Companions [(10), p. 141–146].

### The Recovery and the Strengths Approaches

The recovery approach to mental ill health came to the fore in the late 1990s and has continued to develop since. It postulates that people can lead a meaningful life with and beyond their mental ill health condition (12). This implies that even if some of their symptoms continue to be present, they still can have a meaningful life with psychosocial support, thus calling for an emphasis on care instead of cure. Existing empirical evidence supports this perspective, which has become a formal policy in many countries (13). The unprecedented element of the recovery approach has been the fact that it was initiated by people with the lived experience of mental ill health who rebelled against the prevailing medical model (14), who were joined later by some professionals from all disciplines.

The strengths approach (15) was developed initially in social work, and is by now accepted as an integral component of mental health recovery by all mental health disciplines. It highlights that having difficulties in mental health functioning does not mean losing all abilities, inclusive that of social interaction. The need for a more nuanced assessment of strengths alongside problem areas, the role of motivation to use abilities that have become hidden, and the need to reduce social stigma are emphasised in this approach. Elements of the strengths approach, such as personal efficacy and social capability, are also referred to as social capital (16, 17) which includes all personally owned resources. The role of informal carers, who are usually family members, has also became more central in the changing mental health system (18).

The lessons learned from these conceptual and practice-oriented developments have highlighted the value of experiential knowledge in understanding mental ill health, the impact of specific interventions in this field, the value of mutual support, and the potential of enhanced self-management, alongside learning from scientifically based knowledge which professional providers bring.

The recognition of the positive contribution of experiential knowledge is also exemplified in the development of peer support work in a number of countries (19–21). Peers are people who are utilising their own mental health experiential knowledge as a key component in providing valuable support to other people/peers who experience mental ill health.

### Shared Decision Making (SDM)

SDM entails the contributions of both experiential and scientific knowledge as a method of establishing a process in which key intervention decisions, including medication management as well as psychosocial interventions (8, 22), are reached in mental health practice jointly between experts by experience (i.e. service users) and service providers. Respectful and trusting relationships between service users and providers (23) are necessary conditions for this achievement. SDM calls for attitudinal change by both clinicians and service users, in which sharing experiential knowledge is recognised as a central asset, alongside moving away from the notion that the clinician knows best. Beyond sharing information there is a need for service users to learn to evaluate the information given and to know where to find further information if necessary, as well as to acquire sufficient confidence to present their preferences usefully and convincingly (24). Available decision making aids enable service users and relevant others to understand better the process and to consider their preferred intervention (25).

There are differences in the ways SDM is practiced in the extent to which experts by experience are engaged as co-leading training on SDM and supporting service users in the process of SDM (21, 22, 26, 27) or whether the whole process is led only by clinicians (28).

The stages of SDM in the health system include Choice Talk, Option Talk, and Decision Talk (28, 29), which are practised in both physical and mental health SDM. Decision making sessions are often conducted only between the person/patient and the clinician. However, in care reviews meetings aimed at reviewing the recent past and planning the next phase intervention decisions which take place periodically every 3–6 months, informal carers who are usually family members can be invited too to participate in the decision making process (30).

These stages are reflected also in the FGC process. The choices are part of the initial conversation the co-ordinator has with the people invited to the family meeting as to why such a meeting is necessary, where the key problem areas and the how the FGC process can be of help in resolving them. The options are summarised in the written brief provided by the professional who has initiated the call for an FGC, which every participant at the FGC meeting is given and which are summarised orally by the co-ordinator at the beginning of the meeting. The decision talk is taking place at the FGC meeting in which the participants are asked to opt for a specific option and to follow it up by an action plan as to how it would be implemented.

The main differences between SDM as practised in health systems and the FGC lies in the decision making power given to the key FGC meeting in which clinicians do not participate. But as the options in FGC are prepared by the professional provider, who is also a key figure in the implementation of the decisions made in the FGC, this provider impacts considerably on the option selected by the family. While gaining family support, the individual client has less power in the SDM process as practiced in the FGC than in the one to one meetings between clients and clinicians in the health system SDM process. The one-to-one SDM process is likely to take more than one meeting and hence enables a longer process of establishing trust and respect between clients and professionals.

Currently SDM is not a formally required process in any country, including countries such as the UK where NICE (the National Institute of Excellence) and the DHSC (Department of Health and Social Care) have suggested its use. There is good research evidence that demonstrates the effectiveness of SDM (31, 32), inclusive of cost effectiveness (33, 34). However, the implementation of SDM in mental health is problematic as it requires a considerable attitudinal shift in the views of providers, service users and informal carers (35, 36).

FGC is a strategy in which the family is given the power to exercise shared decision making and an implementation plan within a limited range of options, in collaboration with service providers in the pre-FGC meeting and in the follow up period. Unlike the application of individual SDM, FGC is a legally required practice in all social care agencies which have established it formally as part of their practice.

## Methodological Framework

A scoping narrative review will be provided below. Its inclusion criteria are: Empirical research of FGC; with adults of working age (18–65 years old) experiencing mental ill health; between 2000 and 2020, only in English; both—or either—qualitative and quantitative methodology. The exclusion criteria are: FGC non-empirical research publications; FGC empirical research on children and older people; FGC empirical research not on adults experiencing mental ill health; publications in other languages than English; publications of empirical research before 2000. Articles focused upon in this narrative review are marked with ^*^ in the references list. The literature search included the Scopus database, key social work journals (British Journal of Social Work, European Journal of Social Work, the Family Rights Group^1^, J. of Social Work, Social Work and Social Sciences Review), key mental health journals (Journal of Mental Health Social Inclusion and Mental Health, Mental Health Review), key nursing journals (Issues in Mental Health Nursing, J. of Advanced Nursing, International J. of Mental Health Nursing, Nursing Time), the British Medical Journal (BMJ), Child Abuse and Neglect, J. of Family Law, J. of Sociology and Welfare. The choice of journals was based on the likelihood that they will focus on family Involvement with this client group.

A table summarising the articles focused upon in this narrative review appears in Appendix 1. Two community case studies videos will be looked at for the purpose of illustrating what FGC looks like from the perspectives of the different key players, the processes of reaching shared decisions, outcomes, and the applied evaluation methods. The use of videos has been chosen because they demonstrate well the process of FGC and the emotional experience of FGC from the perspectives of the index client and other family members. Created by the UK based Family Rights Group^1^ (2012), the organisation which promotes FGC practice, the videos are based on real life cases, but do not show specific real people, with participants being depicted through animation. I do not know of any other review of FGC that has used existing videos as part of the research evidence.

A scoping narrative review has been selected because the updated systematic review by Hillebregt et al. (37) highlighted a very small number of studies that fitted the criteria of being conducted with a Randomised control trial (RCT) sampling procedure, which is usually expected to be in place in systematic reviews. The shortage of empirical research on FGC with adults highlights the need to look at how existing research has been conducted, as well as at the gaps in our knowledge of FGC (38, 39). It also raises the question as to whether RCT should be the only criterion for inclusion of research in a systematic review (40). In a recent publication on FGC with adults and the research methods applied to its evaluation in the UK, Manthorpe and Rapaport (41) refer to people experiencing mental ill health, identified as the largest **sub-group** in number within the adult FGC population. They also noted the complexity of evaluating FGC. The only RCT study of adult FGC focusing on people experiencing mental ill health has been carried out in Norway (42–45) is described in the research evidence section below. The two community case studies are based on the analysis of a video of a mother experiencing mental ill health and a video of a daughter's point of view. The videos offer two complementary perspectives of FGC index clients of their expectations from the FGC alongside the decisions made at the family only meeting, and their reflections of the meeting.

### Research Evidence

Most of the existing FGC research focuses on child protection when the key issue is whether the child should be moved from his/her family, and in which the key worker has clear legal duties to be carried out. The key findings highlight considerable satisfaction from the process by the family members and the index client, yet with inconclusive outcomes (46). Some, but not all, studies have a control group. Follow up time varied from one study to another, and most studies did not apply a randomised controlled sampling.

### Existing Systematic Literature Review on FGC With Adults Experiencing Mental Ill Health

Hillebregt et al. (37) have provided the most recent systematic literature review of the key elements and effectiveness of family group decision making interventions in adult health and social care. The three key elements of this review consist of a plan with actions and goals, being family driven, covering three phases which include the follow up of implementation and evaluation [(37), p. 2]. Only studies applying RCT were looked for.

Out of initial 1680 studies, only one met all criteria. Significantly better outcomes in increased social support, mental health and re-employment were demonstrated in the study up to week 23 by the experimental group, but these were not maintained at the end of the follow up year [(37), p. 1]. Conducted by Malmberg-Heimonen et al. (42, 43), it included 149 people with lived experience of mental ill health who have participated in FGC meetings, with the experimental group having an FGC experience, while the control group had treatment as usual. A mixed methods evaluation took place, including filling a questionnaire on social functioning, a mental ill health diagnostic assessment, and the GHQ-12 (General Health Questionnaire) as a measurement of health change at three points in time—prior to the FGC meeting, 3 months later, and 1 year later.

In addition, 15 of the participants were interviewed at the final follow up point (44). The results highlighted a high satisfaction from the FGC process, positive change outcomes at the 3 months follow up, and neutral outcomes at the 12 months follow up in comparison to the outcomes of the control group. The final disappointing results have been explained by the participants as due to lack of reciprocity in social relations within the family and lack of follow up by providers. Attrition in numbers of participants took place too, from 149 to 108, as those who have not completed the FGC full three phases dropped out. The rate of the dropout raises the issue as to whether the RCT sample remained equally randomised at the end as it was at the beginning. Johansen (44) analysed the therapeutic achievements of the 15 interviewees from the experimental group. These included enhancing self disclosure, dialogic communication, and improved family relationships; which are in fact significant achievements for the FGC strategy. It would therefore seem that while not achieving statistical significance, the outcomes for those interviewed have been positive.

### Promising Research

**a. Research on FGC and its impact on social support, resilience and living conditions of index clients**

de Jong and Schout (47), de Jong et al. (48–50), and carried out a large scale follow up research on 41 FGC meetings in terms of their impact on main participants' social support, resilience and living conditions as judged by the index clients, family members, and professionals. Of the total 473 participants (with about 11 participants per each FGC), 312 contributed to the follow up evaluation. A mixed methods evaluation was applied to the multiple case study analysis (51), consisting of interviews and scoring between 1 and 10 of the interview responses. No control group was included in the research design, though there are comments about the index clients who did not complete the evaluation, concerning the likely difficulties that have prevented them from doing so. The FGC meetings were organised by the Public Mental Health Care (PMHC) of Groningen (northern Netherlands) for a client group consisting of people defined as difficult to engage with psychiatric services, experiencing severe mental ill health, addiction, debts, neglected households, and lack of self- care [(50), p. 353]. The FGCs were aimed to enhance the informal support network which could reduce demand for professional care and economic costs, linked to values and conceptual frameworks of creating a more participatory society.

The follow up interviews and scoring of interview thematic content by the researchers took place between 1 and 6 months after the FGC meetings. Participants included index clients (called main participants), family members, the FGC co-ordinators, and professionals. Demographic data is included [(50), Table 2, p. 12].

The results highlighted that the desired change in the three areas of social support, resilience and living conditions, took place in 33 of the 41 cases, reaching statistical significance. The more positive feedback came from the co-ordinators, followed by the clients, with the professionals providing a positive feedback but at a lower level (p. 12). The highest score was given to improvement of social networks, living conditions, and resilience. A small decrease in the wish for further professional care has also been noted.

Unlike other studies, this project demonstrates modest but positive outcomes of the FGC strategy on all three dimensions at the implementation phase, at both the quantitative and the qualitative evaluation facets. It does so with a population experiencing serious difficulties in key living domains, inclusive of mental ill health, many of whom were described as “seemingly hopeless cases” (p. 357). Hence, it indicates that FGC can enable positive impact on key psychosocial dimensions which are critical to success across most living domains. Statistical analysis which included *t*-tests, SPSS 20, and multi-level “nested modelling” analysis demonstrated statistically significant differences between the pre FGC measurements and the post FGC of the key change areas. The study did not aim to cover the degree of change in psychiatric ill health and medical intervention attributed to the FGC intervention.

A number of limitations are noted by the authors, such as having only retrospective reporting, and that not every participant was willing to participate in the evaluation (161 out of the 473 participants). A descriptive design was adopted, rather than an experimental one, the sampling did not follow an RCT model and a control group was not recruited. It is also noted that 30 requests for FGC made by professionals did not lead to an FGC meeting taking place, as the clients invited did not wish to share their problems with their family network, due to feeling ashamed of their situation. It is therefore possible that the finalised sample represents of those who could felt that they could cope with being ashamed about their lives while attending the FGC. It is also possible that the more positive rating by the co-ordinators, as compared with that of the referring professionals, might have been motivated by their greater involvement and motivation to succeed in the FGC.

**b. FGC as a tool to reducing compulsory measures in psychiatric admission**

Compulsory admissions are known to limit the freedom of mental health service users, as well as to making them feel threatened and demeaned, and to curtail exercising personal agency (52, 53).

Schout et al. (54) looked at the conditions in which the use of FGC in reducing compulsory admission is not warranted, through the analysis of 17 cases of compulsory admission, following the multiple case study analysis (51). They concluded that FGC will not be helpful when the client is in acute danger and has difficulties in communicating and making decision. It will also not be helpful when either the professionals working with the index client do not use FGC, or the client and their network are not open to its application. This issue was followed in their 2017 paper (54), as well as by two additional papers led by Mejier et al. (55, 56) which focuses on the contribution of FGC to reducing the use of compulsory measures in mental health settings through the exploration of new partnerships between clients, social networks and professionals (57). Given that most hospitalised psychiatric patients do not stay at the stage of high risk and mental incapacity for long (58) which justify the use of compulsory measures, this is an important issue for further development.

The proposed measures to reduce compulsory admission follow the logic of indirect social engineering, and include the elimination of nursing stations on wards, creating comfort rooms, family rooms, intensive care units, de-escalation of incidents, the deployment of peer supporters, crisis cards and advanced crisis plans, use of the Open Dialogue approach (59) and the Dutch Resource Group Act (RACT) which facilitates involvement in social networks (60). The added value of FGC within this framework is perceived to be focused on the value of family driven decision-making model and a social network strategy which reflect too the indirect social engineering approach. They have found that a one-off FGC may be insufficient to reduce the use of compulsory measures, and that it was applied as a last resort, likely to reduce its effectiveness. The articles by Mejier et al. (55, 56) focused on innovative experimentation of developing promising practice in this complex and demanding area of the mental health system.

**c. FGC effectiveness in reablement in comparison to three other types of family interventions**

Tew et al. (61–63) researched the impact and effectiveness of four types of family interventions, including FGC, with adults experiencing mental ill health in terms of reablement through improved use of personal agency and social interaction. Reablement has been defined as aiming to maximise users' independence, choice and quality of life [(64), p. 4].

The reablement dimensions looked at in this study included personal empowerment and social participation. The four family interventions included systemic family therapy (SFT), behavioural family therapy (BFT), FGC, and the integrated systemic and behavioural approach (ISB). Conceptually this research follows the recovery approach, inclusive of the recovery capital concept (17), and Sen's capability perspective (65).

Methodologically a scoping review was conducted, and Yin's multiple embedded case study approach was applied (66). Twenty-two families were involved; the service user, one family member and one professional per each family were interviewed. Participants filled in scorecards, in which rated positive change in each dimension was calculated by each interviewee on a 5-point Likert scale from 0.5 to 2.5, for the categories of negative change, no change, small change, substantial change, and major change [(66), p. 869]. Positive change was found to be higher for service users and family members than the professionals. Change coincided with starting family meetings close to the time of the mental health crisis experienced by the index client.

Although there was no formal control group, this study provides a comparison of the effectiveness of FGC to reablement to three other models of family work. Outcomes for families participating in FGC were very good concerning social participation and good on personal empowerment [(17), Table 4, p. 17]. Substantial work was required in preparing the FGC meeting to secure that the index client and the family will be in control, which often began while the service user was still in hospital or soon afterwards. FGC was particularly effective in sorting out practical issues rather than in reducing entrenched relationships. FGC and the ISB were more consistently focused on developing the family as a flexible supportive resource. Key elements in this process included the index client being in control, with the family network providing a secure base from which to jump off, rather than to be a safe haven. This study highlights the usefulness of the FGC approach to both policy and practice. According to the authors, the study limitations include that it offers a retrospective subjective perspective and that it is not matched demographically. The small number of families per each family intervention (between 5 and 7 families only) and the lack of follow up to check if the changes continue long term or not were additional limitations.

**d. FGC effectiveness in reducing economic costs**

There is some evidence concerning the economic saving as a result of applying the FGC method, though none is specific to adults experiencing mental ill health challenges. Guthrie (67) mentions a saving of £7,000 per adult FGC in terms of the need to invest less than in a non FGC similar cases, and £77,380 in child FGC, based on a study by Marsh et al (68) published in 1998. Munro et al. (69) in a more updated study outcomes of a Daybreak project show that more of the FGC children stayed at home or with relatives, leading to a considerably lower cost (around £1,598) than children who required a placement outside their family home (around £17,557) [(69), p. 47]. There is no similar evaluation of adult Family Group Conferences.

If cost effectiveness would have been included in FGC evaluation, it would have shed more light on this issue (see further comment on this theme in the Discussion section list of missing issues). However, cost effectiveness analysis, which is taking place in mental health evaluation research (33, 34), does not appear to have been conducted in any of the FGC studies on either children or adults. This analysis would include the calculation of the economic cost, as well as the improvement in quality of life dimensions before and after the FGC meeting and the implementation of its action plan took place. This omission is neither acknowledged, nor explained, in the existing studies.

**e. Community case studies:** **https://www.youtube.com/watch?v=YEDg0FPqGZc**

These two case studies (outlined in Appendix 2) are aimed to provide a thick description (70) of the ambiguous range of thoughts and emotions concerning the forthcoming FGC meeting and its aftermath the mother and daughter portrayed in the videos had.

## Discussion

### Attitudes to Working With Adults Experiencing Mental Ill Health Challenges

The paucity of applying FGC to adults in general, noted by Guthrie (67) as well as by Manthorpe and Rapaport (41), and specifically to adults experiencing mental ill health, requires a further exploration. For example, there are only 10 local authorities in the UK practicing FGC with adults, although the size of the adults of working age population is much larger than that of young people below 18 [(41), Table 2, p. 7]. Is it an indirect statement about less readiness to invest in adults? Is the lower level of readiness to do so due to the belief that less can be achieved with them than with children, or that adults deserve less than children do? Perhaps it is also a statement about the fact that there are more adult in need of complex intervention but fewer workers to meet this need. Guthrie (67) proposes that this is due to the wide variety of the adult population group and the challenge of measuring a wide range of outcomes.

It is also possible that while the key decisions in working with children are whether to move the child away from their family or not to do so, as well as to return the child back to the family, key decisions concerning adults experiencing mental ill health are not so clear cut, with neglect and abuse of the latter being infrequently the highlighted key issues. Given the emphasis in Western culture countries on non-institutionalisation, and the recognition of the possibility of leading a meaningful life without the disappearance of all symptoms subsumed under the heading of the recovery approach for people experiencing mental ill health, SDM is likely to be focused on rehabilitative activities and social interaction alongside the use of medication. Family interventions come to the fore when family relationships are recognised as being problematic and impacting negatively on index clients and family members.

### Methodological Issues

The value of the taken for granted application of RCT as the golden design in the evaluation of FGC is questioned by de Jong et al. (48), de Jong and Schout (71), and Schout (72). They suggest that the complexity of researching FGC requires a highly nuanced approach, which qualitative research methodology would be more suitable for, while acknowledging that a qualitative methodology depends on the interpretative perspective of the researchers. Thus, the assumed suitability of RCT as the best research design is doubted in favour of a framework which will attend to multiplicity, polyvalence and interference, which they call “interplexity” [(71), p. 164]. The complexity of the evaluation of FGC is indeed considerable, and requires further consideration.

The lack of control groups in most of the studies requires further attention too. On the one hand it could be argued that FGC has a comparative dimension from the outset, namely in looking at changes due to experiencing the FGC processes by the index client vs. their family members. On the other hand a comparison with a control group unexposed to FGC while experiencing similar difficulties is likely to provide a more robust comparison. However, having a control group would require a more demanding research design, financial cost, and time.

### Key Findings Concerning the Process of FGC

The research evidence covered above highlights that this group of adult participants in FGC is on the whole satisfied with the process of the strategy and the empowering SDM component it contains, even though the index clients come with a high level of poor self-image, difficult relationships with their families, and of poor social position of long-term duration. This in itself is an indication of the power of the approach to unhook past failures, to lead to at least temporary improvement in these central psychosocial functioning areas, and to enhance the readiness to collaborate better in the future. The indicated potential for economic cost effectiveness—especially in reducing periods of compulsory admissions—is an additional incentive for the systematic use of FGC with this population group.

### Key Outcomes Findings

The outcomes focused upon in the research projects were about psychosocial improvements of the index clients and of increasing their family network both quantitatively and qualitatively. These positive changes have taken place soon after the FGC meeting for most index clients. However, the outcomes of FGC at the follow up stages were less satisfactory than the process, an issue explained by the problematic implementation phase in terms of reduced reciprocal relationships and insufficient contact offered by the providers to the index clients. This phase is crucial for the success of the FGC action plan, yet it is not clear from existing research if it does receive the investment it requires in having a skilled workforce for further work with the index client and their family network.

### Missing Issues

**a. Including shame as an FGC issue**

Including shame as an explicit content and methodological issue seems to be a problem area It has clearly emerged in the FGC video cases, and in the de Jong et al. (49) post FGC evaluation. It is likely to be an issue in other strategies of SDM in mental health, such as periodical care management reviews with informal and formal carers alongside the index client. It appears as an indirect issue in stigma reduction, but it has hardly been addressed either in reflection on research or practice.

**b. Psychiatric Medication Management Issues**

Unlike in individualised shared decision making, where medication management is often discussed, it has not come up in FGC studies of adults experiencing mental ill health challenges. This may be the case due to the lack of involvement of psychiatrists or nurses who are the perceived experts in managing medication, in leading FGC projects with this group. It may be due to the clear preference of FGC researchers to home on psychosocial issues, thus ignoring the significance of psychiatric medication management control for the index client-patient, and for their family members. All too often clients do not wish to take prescribed medication due to the adverse effects of the medication they have been prescribed, to which many carers respond by putting pressure on the clients to take the medication as prescribed, leading to medication compliance becoming a bone of contention in their family relationships. The Israeli based Keshet (Rainbow) training programme offers an example of focusing on providing carers with communication skills that enables the diffusion of the emotional and power struggles between parents and their adult son or daughter who experiences mental illness challenges and leads to more collaborative relationships around this crucial issue (73) which has not reached as yet the agenda of FGC with this client group.

**c. Missing cost effectiveness measurements**

Cost effectiveness analysis, which is taking place in mental health evaluation research (33, 34), does not appear to have been conducted in any of the FGC studies for either children or adults. If applied, this analysis would have provided the calculation of the economic cost, as well as the improvement in quality of life dimensions before and after the FGC meeting and the implementation of its action plan took place. This would have added two central dimensions to the evaluation. This omission is neither acknowledged, nor explained, in the existing studies. It might be reflecting the pre-occupation at this early stage of applying FGC to adults experiencing mental ill health challenges with key psychosocial themes.

d. Missing a thorough impact analysis of barriers and facilitators to the continuation of the achievements reached in the FGC initial meeting across the implementation stage.

## Conclusion

The discussion section has highlighted several achievements of existing empirical studies of applying FGC to adults experiencing mental ill health and their family network. While more good quality studies of the impact of FGC are needed, preferably of mixed methods design led by a qualitative methodological framework, the results of existing FGC with adults experiencing mental ill health are similar to the updated research on FGC with children (46). They indicate that the FGC meeting is experienced positively by all participants, though many of the index clients have had a high number of difficulties and past failures, as an empowering method that opens up communication and support options which have been hitherto blocked, as well as enables SDM to take place. The stumbling point to ensuring the continuation of these achievements seems to be located at the implementation stage. The Discussion section has highlighted several missing issues from the current FGCs agenda. Finally, whether an RCT design should be a must or not in FGC research is a mooted point, as already mentioned above. It seems to me that at this initial stage of evaluating FGC with adults experiencing mental ill health there is a place for more than one approach to the issue of sampling design.

## Author Contributions

SR conceived and wrote the whole article.

## Conflict of Interest

The author declares that the research was conducted in the absence of any commercial or financial relationships that could be construed as a potential conflict of interest.

## Footnotes

^1^https://www.frg.org.uk
