# Supplementary material for: Family Group Conferences as a Shared Decision-Making Strategy in Adults Mental Health Work
Source: Front Psychiatry. 2021 Jul 13;12:663288. doi: 10.3389/fpsyt.2021.663288 (PMC8315278; doi:10.3389/fpsyt.2021.663288)
Supplement: Supplementary file 1 [file Data_Sheet_1.docx]

**Appendix 1**

Table 1 Key articles

| Author/date | Paper type | country | setting | method | analysis |
| --- | --- | --- | --- | --- | --- |
| Barn 2016 | BJSW (British J Social Work) Journal article | UK | FGC | Qualitative  Focus g, profile questionnaire | TA (Thematic analysis) |
| De Jong 2011 | Int. J. of Mental health nursing | The Netherlands | FGC in Public Mental Health | Focus on FGC opportunities - qualitative | Multiple case analysis |
| De Jong 2016 | European J of Social Work | ditto | Ditto+focus on social support and resilience outcomes | Qualitative&Quantitative  (statistics) | Multiple case analysis |
| De Jong 2018 | BJSW | ditto | FGC Procces of change re social support and resilience | Qualitative – focus on the FGC process | Multiple case analysis |
| Hillebregt 2019 | BMJ  Journal article | International | Family group decision making in health and welfare settings | Systematic review, RCT | Systematic analysis |
| Johansen 2014 | BJSW | Norway | FGC process and outcomes | Quali and quant | Mixed methods |
| Johansen 2020 | J. of Family Social Work | Norway | Therapeutic outcomes of FGC | Quali - interviews | TA |
| Malmberg-Heinonen 2011 | BJSW  Journal article | Norway | FGC recipients of welfare assistance with mental ill health | Qualitative and quantitative | TA and statistics |
| Malmberg-H 2013 | EJSW (European J Social Work  Journal article | Norway | Long term effects of FGC with recipients of welfare assistance +mental ill health | Quantitative (standardised measures) and qualitative (interviews) | Statistical analysis and TA |
| Mejier 2017 | Psychiatry J of Advanced Nursing | The Netherlands | Impact of FGC in coercive psychiatry: identity and belonging | Quali thick description and case re-analysis | TA |
| Mejier 2019 | Issues in Mental Health Nursing J | ditto | Ditto + forming new partnerships | Qualt - Thick description and case re-analysis | TA |
| Schout 2017 | Issues in Mental health  Nursing J | The Netherlands | FGC contribution  in compulsory admission  settings | Qualitative case study analysis re FGC as a social network reinforcement | TA |
| Schout 2017 | J of Social Work | The Netherlands | When not to apply FGC in compulsory admission  settings | Qualitative analysis of 17 cases including the index client and professionals | TA |
| Tew 2014 | Family potential centre University report | UK | Whole family approach to Reablement  In mental health | Scoping review | Multiple embedded case study |
| Tew 2015 | NIHR social care school  report | UK | Can whole family approach contribute to reablement | Empirical comparative research qualitative methods | ditto |
| Tew 2017 | BJSW  Journal article | UK | Whole family contributions to reablement | Models, mechanisms, outcomes qualitative comparison of FGC with others whole family models | ditto |
