# Supplementary material for: Family Group Conferences as a Shared Decision-Making Strategy in Adults Mental Health Work
Source: Front Psychiatry. 2021 Jul 13;12:663288. doi: 10.3389/fpsyt.2021.663288 (PMC8315278; doi:10.3389/fpsyt.2021.663288)
Supplement: Supplementary file 2 [file Data_Sheet_2.docx]

**Appendix 2: Community case studies**: <https://www.youtube.com/watch?v=YEDg0FPqGZc>

The two videos which can be viewed via the link above, demonstrate what it is like to be the index

client of a FGC strategy. One of them presents a mother’s perspective, while the other does so

from a daughter/ child’s perspective. The mother’s video had 9,480 viewers, while the child’s video

had 25,454. The child’s video has more humour than the mother’s video, and highlights how a child

can be in control in FGC, with the help of the co-ordinator.

Unlike written case summaries, the videos offer a more nuanced perspective of the emotional ,

visual and body language aspects of FGC, enabling the viewer ( who is an outsider) to learn from

the thick description the main protagonists provide of their contextualised human social action.

The focus on thick description as part of the ethnographic approach comes from Geertz (1973), a

key founder of modern anthropology, as well as from the more recent application of visual

methods in psychology (Reavey, 2021) . Videos – frequently used by now in social media sites - are

recognised as a powerful mode of communication because they combine action and reflection as

well as cognitive and emotional processes, enabling the observer a much richer access to

information than a written account of an interview (Pini and Walkerdine, 2021).

The mother is describing herself as having an unspecified mental illness with depressive features,

turning her incapable to look after her children. The parents are divorced, and the children’s father

is described as living in another country and can’t, or won’t, come to the FGC meeting. Social

services think she cannot look after the children and that her mother/their grandmother cannot do

so either. She dreads the family meeting, ashamed that her “perfect” sister will know what is

happening to her.

The meeting went better than she thought; her sister-in-law and her sister have both offered to

share looking after her children, her doctor came too, and she felt good to feel their support.

The child – a girl age 10-11- says that her mother is ill and bad tempered when unwell. The girl is

often late to school, and forgets things, such as homework. She states that “it is not fun to be late all

the time” .The parents are divorced, and father lives with a new partner and her children. The girl

has to sleep in the living room when she visits her father. Aided by the co-ordinator, she decides

whom to invite, and is happy that she can bring her dog to the meeting, but is afraid the invitees

will either not come, or will quarrel if they come. At the end, the meeting goes much better than

she expected; a plan is made as to who will help her to get to school on time and the father is going

to build her a room for herself in his new home.

Shame and guilt, expressed as dread, characterise the mother’s feelings, while the daughter

expresses anxiety and fear that the people she invited will not come, and confesses to have

“butterflies in her stomach”.

Both the mother and the daughter have been given considerable control in suggesting whom to

invite, and were supported by the co-ordinator throughout the FGC process, but were unsure in

advance what will be the outcomes of the family meeting. Both were relieved with the positive

readiness of the participants to support them without appropriating shame or guilt.

Interestingly, the number of viewers of the girl’s video is three times more than that of the mother’s

video.
